# Hospital Meetings

**Published:** 1902-12-06

**Authors:** 


### HOSPITAL MEETINGS.

### EAST LONDON HOSPITAL FOR CHILDREN.

"Little Known and Little Cared for."

In order to ratify the appointments to the medical staff which had been suggested by the medical committee, a special Court of Governors was held at this hospital on Thursday, November 27th. Two more than the necessary ten were present, and one of these was a lady. Colonel Needham was voted to the chair, and, the appointments having been disposed of, he made a few remarks on the general state of the hospital. The work, he said, was being carried on very well. He was glad to be able to report that King Edward's Hospital Fund had given £1,750 towards the buildings and £100 towards the Convalescent Home. The committee would take immediate steps to make such improvements as were necessary. The grant would not, however, cover the cost; £1,200 was wanted for the laundry and mortuary; but he hoped the friends of the hospital would come to the front as they generally did in the cause of charity. He wished especially to impress upon the Court the necessity of advertising the hospital among their friends. It was very little known, being in a remote part of the town: friends of his own had never heard of it. He did not know what would become of the very poor population were it not for this hospital. It was not backed up by great names, and although his Majesty the King had become a patron the hospital was little known and little cared for.

The meeting, which only lasted a quarter of an hour, closed with a vote of thanks to the chairman.

#### The New Buildings.

The space at the back of the hospital can now show the newly erected laundry, mortuary, and post-mortem room, for which special donations were granted by King Edward's Fund in 1900 and 1901. The laundry is in full work, and is a manifest improvement on the old system of a hand laundry. The cylindrical washer and the circular wringer which wrings the clothes almost dry by means of rapid revolutions are made by a New York firm. The ironing is done by means of gas-heated cylinders and irons, while the drying is effected by means of hot-air cupboards. The entire washing for the hospital is done in the new laundry.

The mortuary and post-mortem room are not quite finished, and are still in the hands of the workmen. The mortuary has a tesselated floor and coloured glass windows, and each shelf is covered by a neat wooden door. The post-mortem room adjoins the mortuary.

A house outside the hospital premises, at a very short distance, has recently been taken for the night nurses. Its bright brass knob and knocker should have a good effect on the other dwellers in a particularly "mean street."

---

### MIDDLESEX HOSPITAL.

Dr. William Caley was voted to the chair at the General Court of Governors on Thursday November 27th.

The Chairman said the purpose of the Court was to transact ordinary business and to consider a proposed alteration in the rule relating to the tenure of the office of chaplain.

Then the secretary-superintendent, Mr. F. Clare Melhado, read the printed précis of transactions of the weekly board, and Sir Arthur Watson proposed its adoption and made a few comments. He said he had a piece of news for the governors which did not appear in the report, viz., that his Majesty the King intended to give an annual contribution of £100 to the hospital, as her late Majesty Queen Victoria had done. In recognition of valuable services rendered during a period of nine years, the governors were invited to appoint their former chairman, Sir Ralph Thompson, K.C.B., to be a vice-president of the hospital. The board learned with profound regret of his continued ill-health, and earnestly hoped that so eminent a colleague might be spared to them.

### Cancer Research.

The speaker then gave some particulars about the investigations into cancer.

The researches are proceeding vigorously. The first volume of the Director's report has recently been published, and has met with a favourable reception from both the President of the Royal College of Physicians and the President of the Royal College of Surgeons, who have signified their appreciation and encouragement of the work.

The second volume will be published early in the new year.

The department is, however, still hampered by want of workers, and the board, acting on the advice of the cancer research committee, have decided to appoint another officer, to be styled junior pathological assistant in the cancer research laboratories. This department has already outgrown the laboratory accommodation provided three years ago, and it has become necessary to arrange for a part of it to be temporarily carried on in the new clinical laboratories; but increased permanent accommodation will have to be considered in the near future.

Through the active interest of Mr. A. Pearce Gould, M.S., several gentlemen have promised to subscribe to the funds for a period of years. Amongst other promises are £250 from Mr. and Mrs. Richard R. Hollins, £100 from E. S. Marcus, Esq., £100 from "Anonymous," £50 from Montagu Lush, Esq., K.C., £50 from Mr. Gould himself, and other smaller amounts. It is estimated that in order to carry on the work efficiently an annual income of £2,000 is required; but it is anticipated that, with the help already received and the assistance of the governors, no difficulty will be experienced in obtaining the necessary funds. With the encouragement thus afforded, and considering the marked advance in the treatment of lupus and other affections by the application of electricity in its various forms, and more especially to the results obtained by this kind of treatment in some cases of cancer, a temporary building in the courtyard of the hospital has been put up. This will be furnished with all the latest and most approved appliances suitable for carrying out this treatment in suitable cases. Mr. C. R. C. Lyster, M.R.C.S., who has had great experience in electrical work as applied to medicine, will be in charge of the department, which will very shortly be opened. The cost of the temporary building amounts to £250.

Three clinical laboratories have also been fitted up and equipped. There is a large general laboratory, which affords bench accommodation for 22 workers, a smaller one which will be set apart for bacteriological work, and the third will be devoted to the chemical investigation of disease. The director is Mr. A. G. R. Foulerton (also director of the cancer research laboratories), and the two pathological assistants are Mr. Leslie Milburn and Mr. C. E. Lakin.

The new operating room attached to the obstetric wards was ready for use on October 1st. There are now 18 beds in these wards, all of which are occupied.

### The Office of Chaplain.

After alluding to the proposed purchase, for the laundry at Hendon, of a steam motor at a cost of £598 10s., Sir Arthur Watson detailed the suggested alteration in the rule governing the tenure of the chaplaincy. The work, he said was very arduous, and the former chaplain had found it too great a strain. The present chaplain, however, the Rev. H. E. Gunson, would, it was hoped, be able to go on for some years yet, and it was thought desirable to alter the rule relating to the giving of notice. He therefore asked that Mr. Gunson might be appointed for two years more (he had already been nearly two years in office), and that at the end of three years he should be allowed to offer himself for reappointment if the board thought fit. The former arrangement was that the chaplain held office only for two years, and could offer himself for only one year in addition.

### The Medical School.

Sir Arthur Watson next proposed certain rearrangements in regard to the medical school. Under the amalgamation scheme, which took effect in 1896, the hospital agreed to advance out of its general funds certain sums required for additional school buildings and their equipment, interest being paid at the rate of £3 per cent. per annum to the general funds out of the income of the school. It was further agreed that the hospital should receive the income arising from the school, and apply it in the first place towards paying all outgoings in respect of the school, including the interest, and then in or towards paying certain specified sums to the unsalaried teaching staff by way of remuneration for their services. The hospital agreed, in the event of the income being insufficient for these purposes, to contribute out of the general funds sums not exceeding £800 in any one year towards making up the deficiency. This agreement has been faithfully adhered to, but has worked out in an unforeseen manner. Owing to the increased expenditure required to keep up the school, nothing has for several years remained wherewith to remunerate the unsalaried teaching staff, and the accounts of the school up to September 30th, 1902, show a deficit of £480 12s. 8d., after crediting them with the annual sums contributed by the hospital in accordance with the agreement. The board regard the medical school as a necessary adjunct to the work of the hospital, and they are of opinion that no part of the teaching should be wholly unremunerated. They have, therefore, given the matter their most careful consideration with a view to preserving and improving the school without laying too heavy a burden on the funds of the hospital, and they have accordingly prepared a revised scheme for the future management of the school.

The main features of the new arrangement are that the school will be more fully recognised as an integral part of the hospital, which will acquire all its property and undertake all its liabilities. The hospital will cease to contribute the sum of £800 a year, and will forego all claim to the sums advanced, or the interest on them, which amounted to nearly £600 a year. The hospital will continue to receive all the income arising from the school, but will devote one-half of all fees, etc., received for teaching towards providing for the whole of the teaching staff. The financial clauses of the scheme will be subject to revision at the end of any financial year.

The new session of the school was opened on October 1st, when Mr. Stephen Paget addressed the students, and Lord Sandhurst distributed the prizes. In the evening a dinner was held at the Trocadero Restaurant, at which Dr. William Cayley presided.

### The Proposals Accepted.

The treasurer, whose lot it is to be in another room counting the money taken from the boxes while the business goes forward, announced that £42 12s. had been put into the boxes in the hospital, and £18 16s. in those outside. One box was temporarily walled up.

Mr. H. E. Ormerod, K.C., in seconding the adoption of the report, said it must have struck many people that the expenditure had been rather unusually large. Whether the money had been well applied was a question for the governors to consider. He could only point to certain departments, inside and outside the hospital, *e.g.*, the extension of the cancer laboratory, which had greatly increased the value of the hospital in scientific research. Then the establishment of the laundry at Hendon had placed more room at the disposal of the hospital. Of course these improvements necessitated more funds. He added that the general interest in hospitals had received a powerful stimulus in consequence of the King's munificence. The meeting, he said, ought not to separate without bearing testimony to the services of the honorary medical and surgical staff, whose time and knowledge were so generously devoted to the patients. The motion was put to the meeting and unanimously passed.

Some reports on matters of administration, such as the fire-extinguishing appliances and the sweeping of the chimneys, were laid on the table, and the meeting separated with a vote of thanks to the chairman.

